# SLC1A2 Variant Is Associated with Essential Tremor in Taiwanese Population

**DOI:** 10.1371/journal.pone.0071919

**Published:** 2013-08-08

**Authors:** Shao-wen Yu, Chiung-Mei Chen, Yi-Chun Chen, Chia Wen Chang, Hong-Shiu Chang, Rong-Kuo Lyu, Long-Sun Ro, Yih-Ru Wu

**Affiliations:** Department of Neurology, Chang Gung Memorial Hospital, Chang Gung University College of Medicine, Taipei, Taiwan; Oslo University Hospital, Norway

## Abstract

Essential tremor (ET), which is one of the most common movement disorders, may lead to severe interference in quality of life. The first genome-wide association study (GWAS) has identified an association of the *LINGO1* variant (rs9652490) with ET in Americans and Europeans. Recently, a second GWAS that was performed in a European population has discovered a new variant (rs3794087) of the main glial glutamate transporter (*SLC1A2*) that increases the risk of ET with an odds ratio of about 1.4. *SLC1A2* encodes for the major glial high-affinity glutamate reuptake transporter in the brain and is a potential ET susceptibility gene. Because replication in a different ethnic population is important for validating a finding, we conducted a case-control study to investigate the *SLC1A2* variant in an Asian cohort with ET in Taiwan. A total of 542 subjects (273 ET patients and 269 controls) were included. The results showed that rs3794087 was associated with ET among the Taiwanese. The odds ratio was 1.37. Our results were similar to those of the second GWAS of ET in Europeans, and this confirms that *SLC1A2* may be a good functional candidate gene for ET. A replication study in another independent population is of importance to validate this association.

## Introduction

Essential tremor (ET), which is one of the most common movement disorders, has a prevalence of 4% after age 40 and 6% after age 65, and it equally affects men and women [1–3]. According to the consensus statement of the movement disorder society on tremor, ET is classically defined mainly by postural tremor of the hands and sometimes of the head [3]. A positive family history has been reported in approximately 50-70% of ET patients in a previous study, and a positive family history for ET is usually associated with a younger age of onset [4,5]. A possible autosomal dominant inheritance pattern with high, but not full, penetrance has been noticed [6–8]. Up to now, three loci have been identified by linkage mapping analysis [9–11], but no definite ET genes have been found [3,4,12,13],

The first genome wide association study (GWAS) for ET found an association of 2 variant sequences (rs9652490 and rs11856808) in the *LINGO1* gene and ET in Europeans and Americans [6,14–16]. A second GWAS study has revealed an association between the single nucleotide polymorphism (SNP) rs3794087 in intron 4 of the main glial glutamate transporter (*SLC1A2*) gene and ET in Germans and Europeans [17]. In order to understand genetic effects across different populations, we aimed to investigate the genetic susceptibilities of the rs1564282 SNP in *SLC1A2* among the Han Chinese in Taiwan population.

## Methods

### Patient population

A total of 273 patients who were diagnosed with probable ET were enrolled. ET was diagnosed according to the Tremor Investigation Group criteria [2]. The inclusion criteria of probable ET are the following: bilateral posture tremor with or without kinetic tremor involving the hands and forearms; tremor that is confined to body parts other than the hands; tremor duration of at least 3 years; and no other neurological deficits. A total of 296 healthy adult volunteers who were enrolled as a control group were matched to the patient group for age, gender, ethnic origin, and area of residence. All subjects gave written informed consents for the study (No 99-0896 A3) under a protocol approved by Chang Gung Medical Foundation Institutional Review Board.

### Genetic analysis

DNA was extracted from leukocytes with standard protocols. The prevalence of the polymorphism rs3794087, which is an intronic variant in SLC1A2, was determined with the polymerase chain reaction (PCR)-restriction fragment length polymorphism method. The sequences of the primers were as follows: 5′-CTGTTGACCTAAGACGTAATGG-3′ (forward primer) and 5′-CCATAGGAAGCAATGCTGAAC-3′ (reverse primer). The PCR was conducted in a reaction containing 100 ng genomic DNA, 0.2 mM of each primer, 0.2 mM dNTPs, 2.0 mM MgCl2, and 0.5 U Taq polymerase. The thermal cycling conditions consisted of 95° C for 4 min, which was followed by 40 cycles of 95° C for 30 s, 55.4° C for 30 s, and 72° C for 30 s. A final extension step of 72° C for 10 min was followed by a 4° C hold cycle. The amplified PCR fragments were digested with HpyCH4III (New England BioLabs, Inc., Ipswich, MA, USA) and separated on a 2.0% agarose gel. The variant (rs 3794087A) eliminate a HpyCH4III restriction site on the PCR product such that such that, upon digestion, a 442-bp fragment appeared instead of 297 and 145 bp fragments, as was the case for the wild type (rs 3794087G).

### Statistical analysis

Chi square tests were used to compare the frequency of the alleles and the genotypes in both cases and controls. Odds ratios (OR) and 95% confidence intervals (CI) were estimated. Statistical significance was defined as *p* values less than 0.05.

## Results

A total of 542 subjects (273 ET patients and 269 normal controls) were included. The mean age at recruitment of the ET patients was 60.3 ± 14.5 years, and the mean age of onset of the ET symptoms was 55.3 ± 16.9 years. Of the ET patients, 47.1% were males, and 25.7% of them had a positive family history of ET. The mean age of recruitment of the 269 controls (47.9% male) was 61.2 ± 13.2 years. The frequencies of the rs3794087 genotypes and alleles of both the ET patients and controls are shown in Table 1. The *SLC1A2* rs3794087 A allele showed a greater frequency in ET patients than in controls (OR, 1.37; 95% CI, 1.02–1.86; *p* = 0.03). After stratifying the results according to the presence of family history, we were unable to find a stronger association in the familial ET group. This may have been due to the small sample size.

**Table 1:** Frequency of Genotype and Allele Polymorphisms of SCL1A2 rs3794087 Among Essential Tremor (ET) and Controls in Taiwan.

	ET (%)	Controls (%)	OR (95% CI)	*P*-value
CC	155 (56.8)	179 (66.5)	1.00	-
CA	113 (41.4)	86 (32.0)	1.5 (1.07–2.16)	0.02
AA	5 (1.8)	4 (1.5)	1.44 (0.35–6.13)	0.6
Common (C) allele	423 (77.5)	444 (82.5)	1.00	-
Minor (A) allele	123 (22.5)	94 (17.5)	1.37 (1.02–1.86)	0.03

P-values and OR calculated in relation to CC genotype and a major allele C

All p-values were calculated by means of chi-squared test.

## Discussion

The first stage of the second GWAS revealed an association between the rs3794087 SNP in intron 4 of the SLC1A2 gene and ET in a German population, and the authors then collected verification samples that included 3 populations (German, Austrian, and Danish origin) in Europe for the second stage of the study. The rs3794087 SNP yielded an OR of 1.43 with a nominal p of 1.16 × 10^-7^ in the combined first- and second-stage samples [17]. Later, Tan et al has reported that the frequency of MAF was lower in ET compared with controls (15% vs 19.5%, OR=0.75, 95% CI 0.60–0.94, p= 0.009) in Singapore’s cohort [18], which was different from the GWAS and our results. Our study found association with the same allele as in the GWAS for ET. The minor allele displayed an OR of 1.37 (p = 0.03; 95% CI, 1.02–1.86). Our results confirmed the finding of GWAS that rs3794087 is a risk variant of ET.

The function of this noncoding SNP is still unknown. The *SLC1A2* gene encodes for the glutamate reuptake transporter. Increased olivary glucose utilization and blood flow in the cerebellum, red nucleus, and thalamus on both sides have been found in positron emission tomography studies of patients with ET [19]. The *SLC1A2* gene is strongly expressed in the inferior olive but not in other structures of the brainstem. In addition, ethanol may increase *SLC1A2* expression and glutamate reuptake activity and significantly reduce tremor in many ET patients [17,19,20].

This study focused on Taiwanese subjects, which definitely have a more homogenous genetic background than Caucasians from different origins. The sample size of our case-control study was modest. There were some limitations of our study. First, only one SNP was analyzed, and it did not exclude an association between other regions within or around the gene. Thus, further investigations, such as whole gene sequencing, should be done to clarify the role of *SLC1A2* in ET. Second, the roles of other genes, gene-environmental interactions, or epigenetic factors have not been evaluated, and these could be confounding variables. Third, there is a different result from another independent Asian cohorts [18], the significance that we observed would likely be more reliable if we were able to perform a meta-analysis across different cohorts.

In conclusion, this is the first report to show association with the same allele as in the GWAS for ET in a Chinese group. We conclude that the SLC1A2 rs3794087 variant contributed to the risk of ET in Taiwan. Further studies in other populations will be useful to validate our finding.

## References

[1] Louis ED (2005) Essential tremor. Lancet Neurol 4: 100-110. doi:10.1016/S1474-4422(05)00991-9. PubMed: 15664542. 15664542 10.1016/S1474-4422(05)00991-9

[2] Deuschl G, Bain P, Brin M (1998) Consensus statement of the Movement Disorder Society on Tremor. Ad Hoc Scientific Committee Mov Disord 13 Suppl 3: 2-23. 9827589 10.1002/mds.870131303

[3] Rowland Lewis P (2010) Merritt’s Neurology. Timothy A, editor. New York.

[4] Deng H, Le W, Jankovic J (2007) Genetics of essential tremor. Brain 130: 1456-1464. doi:10.1093/brain/awm018. PubMed: 17353225. 17353225 10.1093/brain/awm018

[5] Louis ED, Ottman R (2006) Study of possible factors associated with age of onset in essential tremor. Mov Disord 21: 1980-1986. doi:10.1002/mds.21102. PubMed: 16991147. 16991147 10.1002/mds.21102

[6] Stefansson H, Steinberg S, Petursson H, Gustafsson O, Gudjonsdottir IH et al. (2009) Variant in the sequence of the LINGO1 gene confers risk of essential tremor. Nat Genet 41: 277-279. doi:10.1038/ng.299. PubMed: 19182806. 19182806 10.1038/ng.299 PMC3740956

[7] Tanner CM, Goldman SM, Lyons KE, Aston DA, Tetrud JW et al. (2001) Essential tremor in twins: an assessment of genetic vs environmental determinants of etiology. Neurology 57: 1389-1391. doi:10.1212/WNL.57.8.1389. PubMed: 11673577. 11673577 10.1212/wnl.57.8.1389

[8] Lorenz D, Frederiksen H, Moises H, Kopper F, Deuschl G et al. (2004) High concordance for essential tremor in monozygotic twins of old age. Neurology 62: 208-211. doi:10.1212/01.WNL.0000103236.26934.41. PubMed: 14745055. 14745055 10.1212/01.wnl.0000103236.26934.41

[9] Gulcher JR, Jónsson P, Kong A, Kristjánsson K, Frigge ML et al. (1997) Mapping of a familial essential tremor gene, FET1, to chromosome 3q13. Nat Genet 17: 84-87. doi:10.1038/ng0997-84. PubMed: 9288103. 9288103 10.1038/ng0997-84

[10] Higgins JJ, Pho LT, Nee LE (1997) A gene (ETM) for essential tremor maps to chromosome 2p22-p25. Mov Disord 12: 859-864. doi:10.1002/mds.870120605. PubMed: 9399207. 9399207 10.1002/mds.870120605

[11] Shatunov A, Sambuughin N, Jankovic J, Elble R, Lee HS et al. (2006) Genomewide scans in North American families reveal genetic linkage of essential tremor to a region on chromosome 6p23. Brain 129: 2318-2331. doi:10.1093/brain/awl120. PubMed: 16702189. 16702189 10.1093/brain/awl120

[12] Tan EK, Schapira AH (2008) Hunting for genes in essential tremor. Eur J Neurol 15: 889-890. doi:10.1111/j.1468-1331.2008.02226.x. PubMed: 18796073. 18796073 10.1111/j.1468-1331.2008.02226.x

[13] Aridon P, Ragonese P, De Fusco M, Salemi G, Casari G et al. (2008) Further evidence of genetic heterogeneity in familial essential tremor. Parkinsonism Relat Disord 14: 15-18. doi:10.1016/S1353-8020(08)70117-2. PubMed: 17703985. 17703985 10.1016/j.parkreldis.2007.05.002

[14] Thier S, Lorenz D, Nothnagel M, Stevanin G, Dürr A et al. (2010) LINGO1 polymorphisms are associated with essential tremor in Europeans. Mov Disord 25: 717-723. doi:10.1002/mds.22887. PubMed: 20310002. 20310002 10.1002/mds.22887

[15] Jiménez-Jiménez FJ, García-Martín E, Lorenzo-Betancor O, Pastor P, Alonso-Navarro H et al. (2012) LINGO1 and risk for essential tremor: results of a meta-analysis of rs9652490 and rs11856808. J Neurol Sci 317: 52-57. doi:10.1016/j.jns.2012.02.030. PubMed: 22425540. 22425540 10.1016/j.jns.2012.02.030

[16] Deng H, Gu S, Jankovic J (2012) LINGO1 variants in essential tremor and Parkinson’s disease. Acta Neurol Scand 125: 1-7. doi:10.1111/j.1600-0404.2011.01567.x. PubMed: 21470193. 21470193 10.1111/j.1600-0404.2011.01516.x

[17] Thier S, Lorenz D, Nothnagel M, Poremba C, Papengut F et al. (2012) Polymorphisms in the glial glutamate transporter SLC1A2 are associated with essential tremor. Neurology 79: 243-248. doi:10.1212/WNL.0b013e31825fdeed. PubMed: 22764253. 22764253 10.1212/WNL.0b013e31825fdeed PMC3398434

[18] Tan EK, Foo JN, Tan L, Au WL, Prakash KM et al. (2013 ) SLC1A2 variant associated with essential tremor but not Parkinson disease in Chinese subjects. Neurology 80(17): 1618-1619. doi:10.1212/WNL.0b013e31828f1903. PubMed: 23596072. 23596072 10.1212/WNL.0b013e31828f1903

[19] Elble RJ, Deuschl G (2009) An update on essential tremor. Curr Neurol Neurosci Rep 9: 273-277. doi:10.1007/s11910-009-0041-6. PubMed: 19515278. 19515278 10.1007/s11910-009-0041-6

[20] Deuschl G, Bergman H (2002) Pathophysiology of nonparkinsonian tremors. Mov Disord 17 Suppl 3: S41-S48. doi:10.1002/mds.10141. PubMed: 11948754. 10.1002/mds.10141 11948754

